# Economic evaluations considering costs and outcomes of diabetic foot ulcer infections: A systematic review

**DOI:** 10.1371/journal.pone.0232395

**Published:** 2020-04-30

**Authors:** Taylor-Jade Woods, Fisaha Tesfay, Peter Speck, Billingsley Kaambwa

**Affiliations:** 1 College of Medicine and Public Health, Flinders University, Bedford Park, South Australia; 2 College of Science and Engineering, Flinders University, Bedford Park, South Australia; Icahn School of Medicine at Mount Sinai, UNITED STATES

## Abstract

**Background:**

Diabetic foot ulcer (DFU) is a severe complication of diabetes and particularly susceptible to infection. DFU infection intervention efficacy is declining due to antimicrobial resistance and a systematic review of economic evaluations considering their economic feasibility is timely and required.

**Aim:**

To obtain and critically appraise all available full economic evaluations jointly considering costs and outcomes of infected DFUs.

**Methods:**

A literature search was conducted across MedLine, CINAHL, Scopus and Cochrane Database seeking evaluations published from inception to 2019 using specific key concepts. Eligibility criteria were defined to guide study selection. Articles were identified by screening of titles and abstracts, followed by a full-text review before inclusion. We identified 352 papers that report economic analysis of the costs and outcomes of interventions aimed at diabetic foot ulcer infections. Key characteristics of eligible economic evaluations were extracted, and their quality assessed against the Consolidated Health Economic Evaluation Reporting Standards (CHEERS) checklist.

**Results:**

542 records were screened and 39 full-texts assessed for eligibility. A total of 19 papers were included in the final analysis. All studies except one identified cost-saving or cost-effective interventions. The evaluations included in the final analysis were so heterogeneous that comparison of them was not possible. All studies were of “excellent”, “very good” or “good” quality when assessed against the CHEERS checklist.

**Conclusions:**

Consistent identification of cost-effective and cost-saving interventions may help to reduce the DFU healthcare burden. Future research should involve clinical implementation of interventions with parallel economic evaluation rather than model-based evaluations.

## Introduction

Diabetic foot ulcers (DFU) are a common and severe complication of diabetes mellitus characterised by a deep tissue lesion. [1] Factors underlying the development of DFU are peripheral sensory neuropathy, foot deformity, minor foot trauma and peripheral arterial disease. [2] It is estimated that the annual incidence of DFU is 2–4% [3, 4] in developed countries with only two-thirds of cases healing within 12 months. [5] A significant consequence for those DFUs which do not heal is infection which has an incidence of 40.1%. [6] DFU infection is a well-recognised risk-factor for lower extremity amputation which occurs in 8% [5] of cases and carries a 5-year mortality of 74%. [7]

Not only is DFU a source of significant patient suffering, it also brings significant costs to the individual and healthcare system. The cost burden of DFU requires 6 days to 5.7 years of patient income to cover treatment cost with variation based on setting and treatment strategy. [8] The annual cost of DFU treatment is significantly greater than non-diabetic foot ulcer treatment, estimated at $1.38 billion versus $0.13 billion. [9] DFU infection places an additional burden on the healthcare system. Cost per admission among patients with DFU infection versus without was significantly higher in those with infected DFU ($11,290 versus $8,145). [9]

Cost-effective DFU interventions have been identified in previous systematic reviews of economic evaluations, [10–13] however these reviews do not focus on those evaluations where infection is part of the clinical presentation of the DFU or model pathway. Given the high prevalence of infection in DFU and the accompanying economic burden, it is essential to illuminate potentially cost-saving or cost-effective interventions to reduce the burden of DFU infection. We therefore seek to obtain all available economic evaluations that jointly consider the costs and outcomes of DFU with infection considered as part of the clinical situation and critically appraise this literature.

## Methods

### Search strategy

A literature search was conducted using MedLine, CINAHL, Scopus and Cochrane Databases seeking articles published in English from inception to 2020. Terms including diabetic foot, economic evaluation and infection were used as Medical Subject Headings (MeSH) and textwords to capture the outcomes of interest. The MedLine search strategy, adapted for use in other databases, is presented in Table 1. The last database search was 31 Jan 2020.

**Table 1:** MedLine (via Ovid) search strategy adapted for use in other databases.

	Searches
1	Diabetic Foot/
2	Foot Ulcer/ and (diabetes mellitus/ or diabetes mellitus, type 1/ or diabetes mellitus, type 2/)
3	(diabet* adj3 (foot or feet or wound* or ulcer* or lesion* or lower limb* or lower extremit*)).tw,kf.
4	or/1-3
5	economics/ or "costs and cost analysis"/ or "cost allocation"/ or cost-benefit analysis/ or "cost control"/ or "cost of illness"/ or health care costs/ or direct service costs/ or hospital costs/ or health expenditures/ or economics, hospital/ or hospital charges/ or economics, medical/ or fees, medical/ or economics, nursing/ or economics, pharmaceutical/
6	quality-adjusted life years/
7	(QALY or ((cost* or economic*) adj3 (minimi* or utilit* or evaluat* or review* or outcome* or analys* or effect* or benefit))).tw,kf.
8	(cost* or economic*).ti.
9	or/5-8
10	infection/ or community-acquired infections/ or cross infection/ or opportunistic infections/ or superinfection/ or staphylococcal skin infections/ or soft tissue infections/ or suppuration/ or abscess/ or wound infection/ or surgical wound infection/
11	Gangrene/
12	(infection* or abscess* or gangren*).tw,kf.
13	or/10-12
14	4 and 9 and 13
15	Limit 14 to English language

### Study selection

Studies were included if:

1. they compared costs and outcomes in conjunction as part of a stand-alone economic evaluation or alongside a clinical trial or other study design types such as model-based economic evaluations,
2. the study population was exclusively 18 years and over,
3. the study population was diabetic with an infected foot ulcer,
4. they were published in the English language in peer reviewed journals between inception and 2020.

Studies were excluded if:

1. costs and outcomes were not considered and/or compared,
2. study population was not over the age of 18,
3. they were theory papers, letters, editorials, reviews, theses, or dissertations and studies where full texts could not be obtained.

This systematic review was conducted according to the Preferred Reporting Items of Systematic Reviews and Meta-Analyses (PRISMA) guidelines and the checklist is provided as supporting information (S1 Checklist). [14] Articles were identified by screening titles and abstracts, followed by assessment of full-texts for eligibility.

### Data extraction and quality assessment

Key characteristics of the economic evaluations were identified and extracted including study design and perspective, study population, intervention and comparator(s), time horizon and discount rate, methods or model used, costs included, reporting of costs, outcomes measuring health benefit and cost-effectiveness and overall economic evaluation result.

Quality assessment of the reporting of identified studies was performed according to the 24-item Consolidated Health Economic Evaluation Reporting Standards (CHEERS) checklist. [15] Two reviewers independently assessed articles against the criteria, calculating a score out of 24. Each item was assigned one-point, partial marks were awarded if the study did not completely fulfill the criteria, for example if perspective or discount rate choices were not explained. Any differences in marks awarded were discussed by reviewers to reach consensus. Calculation of a percentage score was performed. Given the absence of a largely accepted method for quality appraisal, set categories were based on published literature. [16–19] Studies scoring 85% or higher were of “excellent” quality, studies scoring between 70-<85% of “very good” quality, studies scoring 55-<70% were rated to have “good” quality and studies scoring below 55% were classified as “poor” quality.

## Results

### Study selection

**Fig 1:**
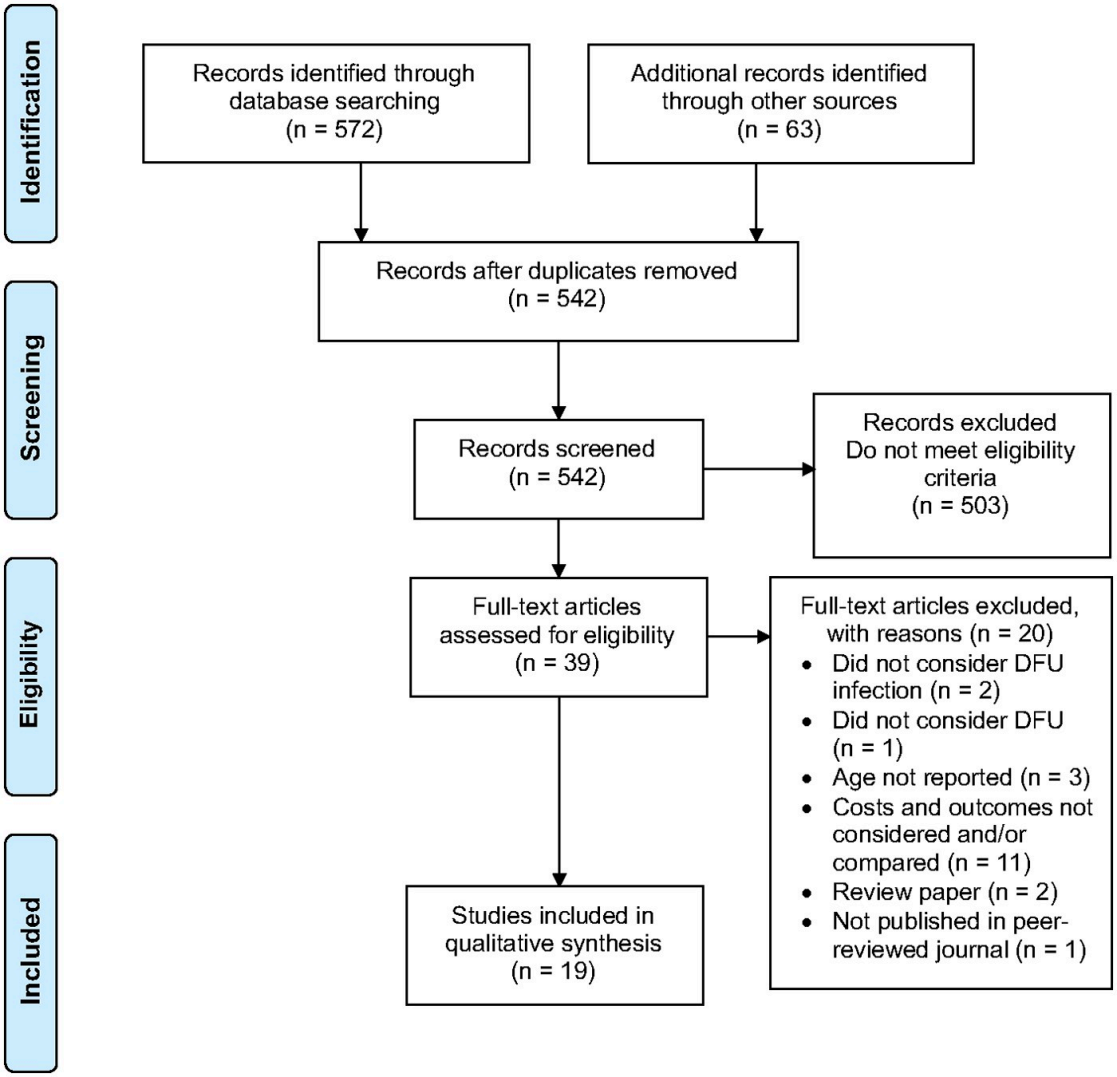
Preferred Reporting Items of Systematic Reviews and Meta-Analyses (PRISMA) flow diagram showing study selection process. DFU: diabetic foot ulcer.

PRISMA guidelines [14] were followed in the study selection process (Fig 1). Database searches identified 527 studies and an additional 63 records were identified through screening of referencing lists; 93 duplicates were removed. Titles and abstracts of 542 articles were screened for eligibility; 503 did not meet the criteria. During this stage, a second reviewer independently assessed 20% of these articles for eligibility and interrater agreement was calculated using Cohen’s kappa statistic. [20] Thirty-nine full-texts were assessed for eligibility. Two papers did not consider DFU infection, [21, 22] three did not report participant age, [23–25] one did not consider DFU, [26] two were review papers [27, 28] and one was not published in a peer-reviewed journal. [29] Eleven papers did not consider and/or compare costs and outcomes as in a full economic evaluation. [30–40] Study eligibility agreement between both reviewers was ‘almost perfect’ with a kappa statistic of 0.83. Nineteen studies were included in the final analysis. All papers [41–59] were full economic evaluations considering and comparing the costs and benefits of interventions against comparators.

### Economic evaluation characteristics

#### Study setting and cohort

Study settings were diverse and accounted for South America, [41, 55] Australia, [42] Canada, [43] China, [59] Europe [47, 49, 51–54, 56] and North America [44–46, 48, 50, 57, 58] (Table 2). Patients across all age groups 18 years and over were captured. Four cohorts were derived from clinical trials [44, 50, 57, 58] and some studies focused on moderate to severe, [48, 55–57] or non-healing [44] DFUs only. DFU infection was a part of the clinical presentation or model pathway in all studies.

**Table 2:** Study results—Summary of economic evaluations of diabetic foot ulcer infections.

Authors, Country, Year	Study design, study perspective	Study population	Intervention, comparator	Time horizon, discount rate	Methods or model	Costs included	Reporting of costs	Measures of health benefits and cost-effectiveness	ICER or Overall economic evaluation result
Cárdenas et al, [41] Peru, 2015	CEA, societal perspective.	Peruvian 18–79 year olds, type 2 diabetics.	Sub-optimal care versus SWC; sub-optimal care versus SWC plus foot temperature monitoring; SWC versus SWC plus foot temperature monitoring.	One year, no discount.	Decision tree model.	Procedural intervention, personnel, medical supplies, examinations and medication. Indirect cost of premature death.	PEN converted to USD based on 2012 exchange rate of 2.64 PEN per USD.	Cost per death averted.	SWC cost saving versus sub-optimal care. SWC plus temperature monitoring cost-effective versus sub-optimal care ICER US$9,405. SWC plus temperature monitoring cost-effective versus SWC ICER US$35,450.
Cheng et al, [42] Australia, 2017	CEA, health system perspective.	Diabetic patients at high risk of DFU, age groups 35–54, 55–74, 75+ years.	SWC versus optimal care.	5 years, 5% discount.	Markov model.	Costs associated with each health state and transition, GP consultation, podiatrist consultation.	2013 AUD.	QALYs.	Optimal care dominant in each age group.
Chuck et al, [43] Canada, 2008	CEA, Ministry of Health perspective.	Canadian 65-year old cohort with DFU, inpatients and outpatients.	Adjunctive HBOT plus SWC versus SWC alone.	12 years, no discount reported.	Decision model based on Guo et al.^43^ applied in Canadian context.	Annual wound care costs adjusted for health states. HBOT costs.	2004 CAD.	Numbers of LEAs, number of healed wounds, number of unhealed wounds.	HBOT plus SWC dominant versus SWC alone.
Dougherty et al, [44] US, 2008	CUA, perspective not reported.	Non-healing DFU patients.	PRP gel versus alternative therapies. Control was saline dressing.	5 years, no discount reported.	Decision analysis model.	Costs per treatment modality, weekly costs of an unhealed ulcer and severe infection. Monthly cost of uncomplicated ulcer. Costs of amputation.	2006 USD.	Clinical outcomes costs and QALYs associated with PRP gel versus alternative therapies.	PRP gel dominant versus saline dressing.
Flack et al, [45] US, 2008	CEA, payer’s perspective.	DFU patients aged 50–65 years.	VAC therapy versus advanced and traditional dressings.	One year, no discount.	Markov model.	Inpatient or outpatient care, professional and pharmacy services, other costs and material dressing.	2006 USD.	Wound healing, incremental cost per QALY	VAC therapy dominant versus advanced and traditional dressings.
Gilligan et al, [46] US, 2015	CMA, third-party payer perspective from Centres for Medicare and Medicaid Services.	Type 1 and 2 diabetics with DFU extending into epidermis.	ECM versus HFDS.	12 weeks, no discount.	Two-state Markov model.	Direct medical costs of care, not itemised.	2014 USD	Cost per wound closure.	ECM cost-saving at US$2,522 versus US$3,889 for HFDS per wound closure.
Guest et al, [47] UK, 2018	CEA, perspective not reported.	130 DFU patients.	SWC alone versus SWC plus a collagen-containing dressing.	Four months, no discount.	Decision model.	Wound care (dressing costs, bandages).	Patient management under National Health Services estimated at 2015/16 prices in GBP.	QALYs gained after 4 months.	Collagen-containing dressing plus SWC dominant versus SWC alone.
Guo et al, [48] US, 2003	CUA, payer’s and societal perspectives.	1,000 60-year-old severe DFU patients (Wagner grade 3 and above).	SWC plus HBOT versus SWC alone.	Time horizon 1, 5 and 12-years. 3% discount.	Decision tree model.	HBOT technical and physician fees. Amputation surgery, inpatient care, rehabilitation, first-year outpatient visits.	2001 USD.	LEAs averted, QALYs gained.	Adjunct HBOT cost-effective. ICER at years 1, 5 and 12 were $27,310, $5,166, and $2,255 respectively.
Lobmann et al, [49] Germany, 2018	CEA, German statutory health insurance.	240 DFU patients at mean age of 64 years.	TLC-NOSF dressing versus control dressing.	20 weeks, 100 weeks; discount not reported.	Markov model.	Nursing, medical consultation/ physician fees, wound care products, inpatient stay and pharmacotherapy.	Euro, currency year not reported.	Wound healing rate.	TLC-NOSF dominant versus control dressing.
McKinnon et al, [50] US, 1997	CMA, hospital perspective.	DFU patients enrolled in a completed randomised double-blind trial.	Ampicillin/sulbactam versus imipenem/cilastatin.	Time horizon not explicit, intervention duration or until clinical failure. No discount.	Decision model.	Antibiotic acquisition, preparation and administration, hospital bed. Treatment after clinical failure. Treatment of adverse events.	1994 USD.	Treatment success, failure, indeterminate.	Success rate identical for both interventions, ampicillin/sulbactam cost-saving ($14,084 vs. $17,008).
Ortegon et al, [51] Netherlands, 2004	CUA, no specific perspective.	10,000 newly-diagnosed type 2 diabetics in the Netherlands.	IGC alone, GFC alone, IGC plus GFC.	Time horizon not specified, 3% discount.	Risk-based Markov model.	Labour, medication, laboratory costs, materials and procedure costs.	1999 USD.	QALYs.	IGC plus GFC <25,000 per QALY. IGC alone was $32,057 per QALY. GFC alone ranged from $12,169 to $200,100.
Persson et al, [52] Sweden, 2000	CEA, perspective not reported.	Neuropathic DFU patients treated with GWC.	Becaplermin gel plus GWC versus GWC alone.	12, 18 and 24 months. Costs are discounted at 5% per year.	Markov simulation model.	Topical treatment, antibiotics, outpatient and inpatient care, social services/home care, amputation and prothesis.	1999 USD.	Ulcers healed or healing time.	Becaplermin plus GWC dominant versus GWC alone.
Redekop et al, [54] Netherlands, 2003	CEA, societal perspective.	Patients enrolled in the Apligraf Diabetic Foot Ulcer Study (ADFUS).	Apligraf plus GWC versus GWC alone.	12 months, no discount.	Markov-based simulation model.	Apligraf costs, outpatient clinic visits, podiatrist visits, GP visits, homecare, hospital days, debridement, antibacterial, diagnostic tests, footwear and dressings.	1999 Euro.	Incremental cost per ulcer-free month gained and per amputation avoided.	Apligraf plus GWC dominant versus GWC alone.
Romero Prada et al, [55] Colombia, 2018	CEA, Colombian health system perspective.	Patients diagnosed with Wagner’s grade 3 or 4 DFU.	rhEGF plus SWC versus SWC alone.	5-year horizon, 5% discount.	Markov model.	Direct costs of health resources, procedures, prothesis, rehabilitation and inputs.	2016 and 2017 USD.	QALYs.	rhEGF cost effective, ICER US$13,428.
Ragnarson et al, [53] Sweden, 2001	CUA, perspective not reported.	10,000 diabetics over the age of 24.	Optimal prevention versus current prevention strategies in Sweden.	5 years, 3% discount.	Markov model.	Ulcer and amputation prevention, amputations, home care and social services, costs of remaining in or transitioning between health states, prostheses, inpatient and outpatient care.	Costs inflated to 1998 SEK, converted to Euro.	QALYs.	Optimal prevention was dominated in group 1. Optimal prevention dominant in all risk groups except group 3 in age groups 24–69 (€5087/QALY) and 70–84 (€4045/QALY).
Tesar et al, [56] Slovakia, 2017	CUA, perspective of health care payers.	Not reported.	Heberprot-P plus GWC versus GWC alone.	5- and 10-year time horizons, 5% discount.	Markov model.	Heberprot-P treatment cost. No other cost inclusions reported.	2011 Euro.	QALYs.	Heberprot-P plus GWC dominated by GWC alone.
Tice et al, [57] US, 2007	CMA, hospital perspective.	Adult diabetics previously enrolled in clinical trial with moderate to severe foot infections.	Intravenous ertapenem versus piperacillin plus tazobactam.	Time horizon not reported. Discount not reported.	Cost comparison between antibiotic regimens.	Drug acquisition, preparation, labour costs, consumable costs. Supply costs discounted by 40%.	2005 USD.	N/A.	Ertapenem cost saving versus piperacillin/tazobactam ($355.55 versus $502.76).
Waycaster et al, [58] US, 2016	CEA, third-party payer perspective.	DFU patients enrolled in one of three phase III clinical trials.	Becaplermin gel plus GWC versus GWC.	1 year, no discount.	WSA reduction rate used to predict costs and outcomes of wound healing.	Becaplermin gel, patient evaluation and management, procedure cost, ankle brachial index test.	2013 USD.	Cost per 1cm^2^ reduction in WSA.	Becaplermin plus GWC dominant versus GWC alone.
Wu, et al, [59] China, 2018	CEA, Chinese healthcare system.	Patients with type 2 diabetes at low, moderate ang high risk of DFU.	Optimal care versus SWC.	5 years, 5% discount.	Decision–analytic model.	Direct medical costs and resource utilisation.	2016 USD.	QALYs.	Optimal wound care dominant versus SWC.

CEA, cost-effectiveness analysis; SWC, standard wound care; IDF, International Diabetes Federation; PEN, Peruvian Nuevos Soles; USD, United States dollar; AUD, Australian dollar; CAD, Canadian dollar; CMA, cost-minimisation analysis; CUA, cost-utility analysis; ICER, incremental cost-effectiveness ratio; HBOT, hyperbaric oxygen therapy; DFU, diabetic foot ulcer; PRP, platelet rich plasma; LEA, lower extremity amputation; VAC, vacuum assisted closure; ECM, extracellular matrix; HFDS, human fibroblast-derived dermal substitute; GBP, British pound sterling; TLC-NOSF, technology lipido-colloid sucrose octasulfate or nano-oligosaccharide factor; IGC, intensive glycemic control; GFC, good foot care; GWC, good wound care; WSA, wound surface area; rhEGF, recombinant human epidermal growth factor; SEK, Swedish krona; OPAT, outpatient parenteral antimicrobial therapy. papers. [44–46, 49] Standard dressings were a comparator to advanced dressings or vacuum-assisted closure (VAC) therapy [45] or technology lipido-colloid sucrose octasulfate or nano-oligosaccharide factor (TLC-NOSF) dressings. [49] Dougherty et al. [44] used a saline dressing as a control when comparing to platelet-rich plasma (PRP) gel and Gilligan et al. [46] compared extracellular matrix (ECM) and human fibroblast-derived dermal substitute (HFDS) dressings. McKinnon et al. [50] and Tice et al. [57] directly compared two antibiotics, one of which was considered conventional care, but neither study used the same antibiotics.

#### Study perspectives

The economic perspective taken by each study determines the cost and benefits included. [17] The societal perspective was taken by Cárdenas et al. [41] and Redekop et al. [54] The perspective of the healthcare system was taken in six studies [42, 43, 50, 55, 57, 59] and the payers perspective was taken in five [45, 46, 49, 56, 58] (Table 2). Both the societal and payers perspectives were taken by Guo et al. [48] Perspective was not reported in five studies, [44, 47, 51–53] however Ortegon et al. [51] discussed some results from the policy and clinical perspective.

#### Intervention and comparator

A multitude of interventions were used to manage DFUs, typically antimicrobials or wound care strategies, reflecting components of the standard treatment of DFUs [2, 60] (Table 2). Most economic evaluations assessed adjuncts to standard wound care strategies. [41, 47, 54–56] Two studies assessed becaplermin gel plus good wound care (GWC) [52, 58] and two studies assessed hyperbaric oxygen therapy (HBOT). [43, 48] The overall wound care strategy was compared in three studies, each comparing what was locally considered as standard versus optimal wound care. [42, 53, 59] Wound dressings were assessed in four.

#### Time horizon

Time horizons should be specified and cover the provision of the intervention and tracking of costs and consequences/benefits. Ideally, they should reflect actual clinical practice. One-year [41, 45, 54, 58] and five-year time horizons were most common. [42, 44, 53, 55, 56, 59] The longest time horizon was 12-years by Chuck et al. [43] Short time horizons were taken by Gilligan et al. [46] and Guest et al. [47] of three and four months respectively. Two time horizons were taken in two papers, Tesar et al. [56] used 5- and 10-year time horizons and Lobmann et al. [49] used 20-week and 100-week time horizons. Persson et al. time horizon was between one and two years. [52]

Four studies did not explicitly report the time horizon. [48, 50, 51, 57] In McKinnon et al., [50] time horizon was ambiguous, reported as being from the beginning of study-drug commencement to the completion of study-drug or secondary treatment following clinical failure. Overall, reported time horizons ranged from 12 weeks to 12 years.

#### Discount rates

Discount rates allow economic evaluations to account for changes in the value of money over time. Four studies did not report a discount rate. [43, 44, 49, 57] Three studies used a 3% discount rate, however none of them justified this choice. [48, 51, 53] Four studies used a 5% discount. [42, 52, 55, 59] All studies with a time horizon of one year of less did not discount costs. [41, 45–47, 50, 54, 58]

#### Study designs and models used

Eleven studies were CEAs, [41–43, 45, 47, 49, 52, 54, 55, 58, 59] another five were CUAs, [44, 48, 51, 53, 56] while three studies found the interventions were equally efficacious, therefore costs were directly compared as in a CMA. [46, 50, 57]

Two studies did not use an economic model to simulate the impact of interventions on DFU. [57, 58] Tice et al. performed a direct cost comparison as each intervention was assumed to be equally efficacious. [57] Waycaster et al. used wound surface area reduction rates to predict costs associated with DFU healing. [58] Seven studies used a decision-tree analytical model. [41, 43, 44, 47, 48, 50, 59] The Markov model was the most common choice and was used in ten studies. [42, 45, 46, 49, 51–56]

#### Costs included

There was variation between cost inclusions in each study depending on setting, perspective and interventions investigated. Some studies kept a narrow scope of direct costs associated with the intervention [47, 56, 57] or ulcer state, [44] but most studies had a broad scope of inclusions that captured DFU intervention, rehabilitation and patient management. [41, 48, 49, 52–55, 58, 59] Indirect costs were only itemised in one study. [41]

#### Overall economic evaluation results

All evaluations except one concluded the intervention assessed was cost-effective or cost saving. This means all interventions provided more health benefit at a lower incremental cost most of the time. All CMAs showed the intervention achieved equal health benefit at lower costs versus the comparator.

While the evaluations are incomparable due to heterogeneous methods and analyses, the intervention dominated the comparator in nine studies by providing greater health benefits at lower cost. [42–45, 47, 49, 52, 54, 59] The intervention was dominated by the comparator in one study due to the unit cost of the adjunct. [56] Six studies found the intervention to be cost-effective [41, 48, 51, 53, 55, 58] and three found the intervention was cost saving as the health benefits were equivalent. [46, 50, 57] Ragnarson et al. [53] found the intervention was dominant or cost-effective for higher risk patients in all age groups, but was dominated by the comparator in the lowest risk group.

Adjuncts were dominant or cost-effective interventions in nine of ten papers. McKinnon et al. [50] and Tice et al. [57] evaluated antimicrobials, both finding the intervention was cost saving. In the four studies which assessed dressings, three found the intervention was dominant [44, 45, 49] and one found the intervention was cost-saving. [46] Three papers compared standard wound care to optimal wound care strategies. [42, 53, 59] Cheng et al. [42] and Wu et al. [59] found optimal wound care was dominant while Ragnarson et al. [53] found optimal wound care was dominant or cost-effective only in higher risk groups.

### Quality assessment of economic evaluations

The reporting quality of each paper was assessed against the 24-item CHEERS checklist. [15] Studies were allocated one mark for each criterion met in full (represented by √), 0.5 marks if the criterion was partially met (represented by ≠) or if the criterion was not met, 0 marks (represented by ×) (Table 3). The total possible score was reduced by one point for all criteria that were not applicable (N/A) to a single study. For example, studies that were not model-based could not be assessed by criteria 15 or 16 (model justification and assumptions). Six studies were of “excellent” quality (scoring >85%). [41, 45, 49, 55, 58, 59] Nine studies were of “very good” quality (scoring 70-<85%) [42, 43, 46–48, 50, 52, 54, 56] and four studies were of “good” quality (scoring 55-<70%). [44, 51, 53, 57] The best addressed criterion was findings and limitations; conversely, the least addressed areas were study perspective, time horizons and discount rates. Many studies failed to report these and where reported, justification of their relevance was absent. Similarly, discussion of choice of outcomes was rarely related to the particular health state or intervention.

**Table 3:** Quality assessment of publications against the Consolidated Health Economic Evaluations Reporting Standards (CHEERS) checklist.

	Title identified as economic evaluation	Structured abstract	Intro provides context, study question and relevance	Population	Setting and location	Study perspective	Comparators	Time horizon	Discount rate
**Authors, year**	**1**	**2**	**3**	**4**	**5**	**6**	**7**	**8**	**9**
Cárdenas, et al 2015	√	√	√	√	√	√	√	√	√
Cheng et al. 2017	√	√	√	√	≠	√	√	√	√
Chuck et al. 2008	√	√	√	√	√	√	√	√	≠
Dougherty et al. 2008	√	≠	√	≠	×	×	≠	≠	×
Flack, et al 2008	√	√	√	√	√	√	√	√	×
Gilligan et al 2015	√	√	√	√	√	√	√	√	×
Guest et al 2018	√	√	√	≠	√	×	√	√	√
Guo et al. 2003	√	≠	√	≠	√	√	≠	√	≠
Lobmann, et al 2019	√	√	√	√	√	√	√	√	×
McKinnon et al. 1997	√	≠	√	√	√	√	≠	≠	√
Ortegon et al. 2004	≠	≠	√	√	√	×	√	×	≠
Persson, et al 2000	√	√	√	×	√	√	√	√	√
Ragnarson Tennvall et al. 2001	≠	≠	√	√	≠	×	≠	≠	√
Redekop et al. 2003	√	√	√	≠	≠	≠	√	≠	×
Romero Prada, et al 2018	√	√	√	×	√	√	√	√	√
Tesar, et al. 2017.	√	√	√	×	√	√	√	√	√
Tice et al. 2007	√	≠	√	√	≠	≠	≠	×	×
Waycaster et al. 2016	√	≠	√	≠	√	√	√	√	√
Wu, et al 2018	√	√	√	≠	≠	√	√	√	√
	**Outcomes and relevance**	**Measurement of effectiveness**	**Preference-based outcomes**	**Resources and costs estimation**	**Currency, date, and conversion**	**Model justification**	**Model assumptions**	**Analysis methods**	**Parameters of values**
**Authors, year**	**10**	**11**	**12**	**13**	**14**	**15**	**16**	**17**	**18**
Cárdenas, et al 2015	√	√	N/A	√	√	√	√	√	≠
Cheng et al. 2017	√	√	N/A	√	√	√	≠	≠	≠
	**Outcomes and relevance**	**Measurement of effectiveness**	**Preference-based outcomes**	**Resources and costs estimation**	**Currency, date, and conversion**	**Model justification**	**Model assumptions**	**Analysis methods**	**Parameters of values**
**Authors, year**	**10**	**11**	**12**	**13**	**14**	**15**	**16**	**17**	**18**
Chuck et al. 2008	≠	√	N/A	√	√	≠	≠	≠	√
Dougherty et al. 2008	≠	≠	√	≠	√	≠	√	≠	≠
Flack, et al 2008	√	√	N/A	√	√	√	√	√	√
Gilligan et al 2015	≠	√	N/A	√	√	√	√	√	√
Guest et al 2018	√	√	N/A	√	√	√	√	√	√
Guo et al. 2003	≠	√	√	≠	√	≠	√	≠	√
Lobmann, et al 2019	√	√	N/A	√	√	√	√	√	√
McKinnon et al. 1997	√	√	N/A	√	√	≠	×	≠	≠
Ortegon et al. 2004	≠	≠	√	√	≠	√	×	≠	√
Persson, et al 2000	√	√	N/A	√	X	≠	√	√	√
Ragnarson Tennvall et al. 2001	≠	×	√	≠	√	√	≠	≠	√
Redekop et al. 2003	≠	≠	N/A	√	√	√	√	≠	√
Romero Prada, et al 2018	√	√	N/A	√	√	√	√	√	√
	**Outcomes and relevance**	**Measurement of effectiveness**	**Preference-based outcomes**	**Resources and costs estimation**	**Currency, date, and conversions**	**Model justification**	**Model assumptions**	**Analysis methods**	**Parameters of values**
**Authors, year**	**10**	**11**	**12**	**13**	**14**	**15**	**16**	**17**	**18**
Tesar et al. 2017	√	√	≠	√	×	√	√	≠	×
Tice et al. 2007	≠	≠	N/A	√	√	N/A	N/A	√	√
Waycaster et al. 2016	√	√	N/A	√	√	N/A	N/A	√	√
Wu, et al 2018	√	√	N/A	≠	≠	√	√	√	√
	**Incremental costs**	**Sensitivity of incremental costs or model sensitivity analysis**	**Heterogeneity explanation**	**Findings and limitations**	**Funding source(s)**	**Conflict of interest**	**Total**	**%**	
**Authors, year**	**19**	**20**	**21**	**22**	**23**	**24**			
Cárdenas, et al 2015	√	√	≠	≠	√	√	21.5/23	93%	
Cheng et al. 2017	≠	≠	≠	√	√	√	19/23	82%	
Chuck et al. 2008	≠	≠	√	√	×	×	17.5/23	76%	
Dougherty et al. 2008	√	√	√	√	≠	√	15.5/24	65%	
Flack, et al 2008	√	√	√	√	×	×	20/23	87%	
	**Incremental costs**	**Sensitivity of incremental costs or model sensitivity analysis**	**Heterogeneity explanation**	**Findings and limitations**	**Funding source(s)**	**Conflict of interest**	**Total**	**%**	
**Authors, year**	**19**	**20**	**21**	**22**	**23**	**24**			
Gilligan et al 2015	≠	√	≠	√	×	×	18.5/23	80%	
Guest et al 2018	√	√	≠	√	×	×	19/23	82%	
Guo et al. 2003	√	√	√	√	×	×	18/24	75%	
Lobmann, et al 2019	√	√	√	√	×	√	21/23	91%	
McKinnon et al. 1997	√	≠	≠	√	√	×	17/23	74%	
Ortegon et al. 2004	√	≠	×	√	×	×	14/24	58%	
Persson, et al 2000	×	√	≠	≠	≠	≠	17.5/23	76%	
Ragnarson Tennvall et al. 2001	√	≠	√	√	√	×	16/24	67%	
Redekop et al. 2003	√	√	≠	√	√	×	17/23	74%	
Romero Prada, et al 2018	√	√	≠	√	√	√	21.5/23	93%	
Tesar, et al 2017	√	√	≠	√	×	×	17.5/24	73%	
	**Incremental costs**	**Sensitivity of incremental costs or model sensitivity analysis**	**Heterogeneity explanation**	**Findings and limitations**	**Funding source(s)**	**Conflict of interest**	**Total**	**%**	
**Authors, year**	**19**	**20**	**21**	**22**	**23**	**24**			
Tice et al. 2007	√	×	√	√	×	√	14/21	66%	
Waycaster et al. 2016	√	≠	√	√	×	×	18.5/21	88%	
Wu, et al 2018	√	√	√	√	√	√	21.5/23	93%	

√: criteria met fully; ×: criteria not met; ≠: criteria partially met; N/A: not applicable.

## Discussion

Whilst the need for effective DFU interventions increases, few have been subject to economic evaluation. All interventions examined in these evaluations were cost-effective or cost-saving in a clinical situation involving DFU infection. Collectively, they suggested that short- and long-term implementation of such interventions could reduce the burden of DFU infection on healthcare systems while providing optimal patient management. Although the evaluations captured the standard care for DFUs and associated costs, other considerations were made on the issue. These included assessments of antibiotic efficacy, route and setting of administration and the overall strategies embodied in a variety of guidelines and recommendations.

Seventeen evaluations were model-based and did not implement the intervention in clinical settings. [41–56, 59] They relied on published data and literature to build a model that simulated the intervention and transitions between health states. Decision tree and Markov models were used, and model justification and assumptions were generally well-reported. Although models are widely accepted methods for informing policy-making decisions, [19] future research would benefit from implementing the interventions in a clinical setting.

Together all evaluations assessed at least one component of the DFU management strategy, with adjuncts assessed in most papers. Because the treatment of DFUs has multiple components including antimicrobials and standard wound care, it was to be expected that these economic evaluations would be too heterogeneous for comparison.

Quality assessment of studies against the CHEERS checklist [15] found all studies to be of “excellent”, [41, 45, 49, 55, 58, 59] “very good” [42, 43, 46–48, 50, 52, 54, 56] and “good” [44, 51, 53, 57] quality, adding strength to the conclusions drawn in this review. Furthermore, some studies included were published before the conception of the CHEERS checklist in 2013. [15] This shows that high-quality studies with earlier publication dates conform with the guidelines crystallised in the checklist and supports the comprehensiveness of the search strategy.

There are some limitations to our study. The results of this review rely exclusively upon studies published in English, which may not represent all research. Grey literature that is unpublished or published but non-commercially available was not searched due to time constraints. Full texts that could be obtained were limited to those accessible by the Flinders University library system. Although few studies were parallel evaluations to clinical trials, the majority of studies were model-based so the results relied heavily upon simulation of intervention effects rather than clinical application.

## Conclusion

In conclusion, economic evaluations have considered all aspects of DFU intervention, finding there is potential to select more cost-saving and cost-effective alternative to reduce the burden of DFU. Instead of model-based evaluations, future research should be directed toward actual implementation of interventions in clinical settings with economic evaluations in parallel.

## Supporting information

S1 Checklist Preferred Reporting Items of Systematic Reviews and Meta-Analyses (PRISMA) checklist. (DOCX)
